# Supplementary material for: Enzyme-constrained metabolic model of Treponema pallidum identified glycerol-3-phosphate dehydrogenase as an alternate electron sink
Source: mSystems. 2025 Apr 7;10(5):e01555-24. doi: 10.1128/msystems.01555-24 (PMC12097511; doi:10.1128/msystems.01555-24)
Supplement: Text S1 — GAM derivation and supplemental figures. [file msystems.01555-24-s0001.docx]

**Enzyme-constrained Metabolic Model of *Treponema pallidum* Identified Glycerol-3-phosphate Dehydrogenase as an Alternate Electron Sink**

Nabia Shahreen^1, †^, Niaz Bahar Chowdhury ^1, †^, Edward Stone^1^, Elle Knobbe^1^, Rajib Saha^1^

^1^Department of Chemical and Biomolecular Engineering, University of Nebraska-Lincoln

^†^These authors contributed equally.

Running title: Enzyme-constrained metabolic model of *T. pallidum*

^*^Address correspondence to Rajib Saha, rsaha2@unl.edu

**Supplementary File**

**Supplementary Text**

To derive the new substrate uptake equation for *T. pallidum*, we began by examining Pritt’s equation for *P. putida*, $q = 10\mu+ 0.077$ (1). Here q is the specific glucose uptake rate and $\mu$ is the specific growth rate. This equation was constructed using specific parameters, including the maintenance coefficient (m) and the maximum protein yield ($Y_{G}$) for *P. putida*. The original values for *P. putida* assumed a maintenance coefficient of $0.023 g glucose/g protein/h$ and a protein yield of $1/3 g protein/g glucose$ for a protein content of 60%.

First, to adjust the maintenance coefficient for *T. pallidum*, we converted the original maintenance coefficient to a dry cell weight (DCW) basis by dividing by the protein content $(0.6)$, resulting in $mDCW = 0.0383 g glucose/gDCW/h$. To reflect the conditions of $70\%$ protein content, this value was further adjusted by multiplying by 0.7/0.6, yielding $mDCW = 0.0447 g glucose/gDCW/h$.

Next, we adjusted the maximum protein yield ($Y_{G}$). The original yield for $60\%$ protein content was $1/3 g protein/g glucose$, which, when converted to a dry cell weight basis, corresponded to $1/Y_{GDCW}$ $= 10 mmol/gDCW$. For the 70% protein content, we scaled this value using a factor of 0.6/0.7, resulting in $1/Y_{GDCW}= 8.57 mmol/gDC$. Combining these adjusted parameters into the original equation format, led to the new expression: $q = 8.57\mu+ 0.0447$.

In parallel, to calculate the growth-associated maintenance (GAM), we calculated $\mu$ from the Pritt’s equation with a glucose uptake rate from $1 mmol/gDCW/hr$ to $10 mmol/gDCW/hr$. We did the same from iTP251. Then we calculated root mean square error (RMSE) between the iTP251 predicted growth and Pritt’s equation predicted growth. We continued calculating the RMSE for different values of GAM, until iTP251 faces infeasibility. After the $48.69 mmol/gDCW/hr$ GAM value, iTP251 became infeasible (Table S2). Thereby, we picked $48.69 mmol/gDCW/hr$ as the GAM value.

For NGAM calculations, we set up the glucose uptake rate in iTP251 as $0.0447 mmol/gDCW/hr$, and maximized the ATP maintenance reaction (rxn05145). The maximum value of the ATP maintenance reaction was $1.5 mmol/gDCW/hr$. Thereby, we used that value as NGAM. The optimization problem can be accessed in the GitHub repository (https://github.com/ssbio/Treponema_pallidum_Nichols).

**Supplementary Figures**


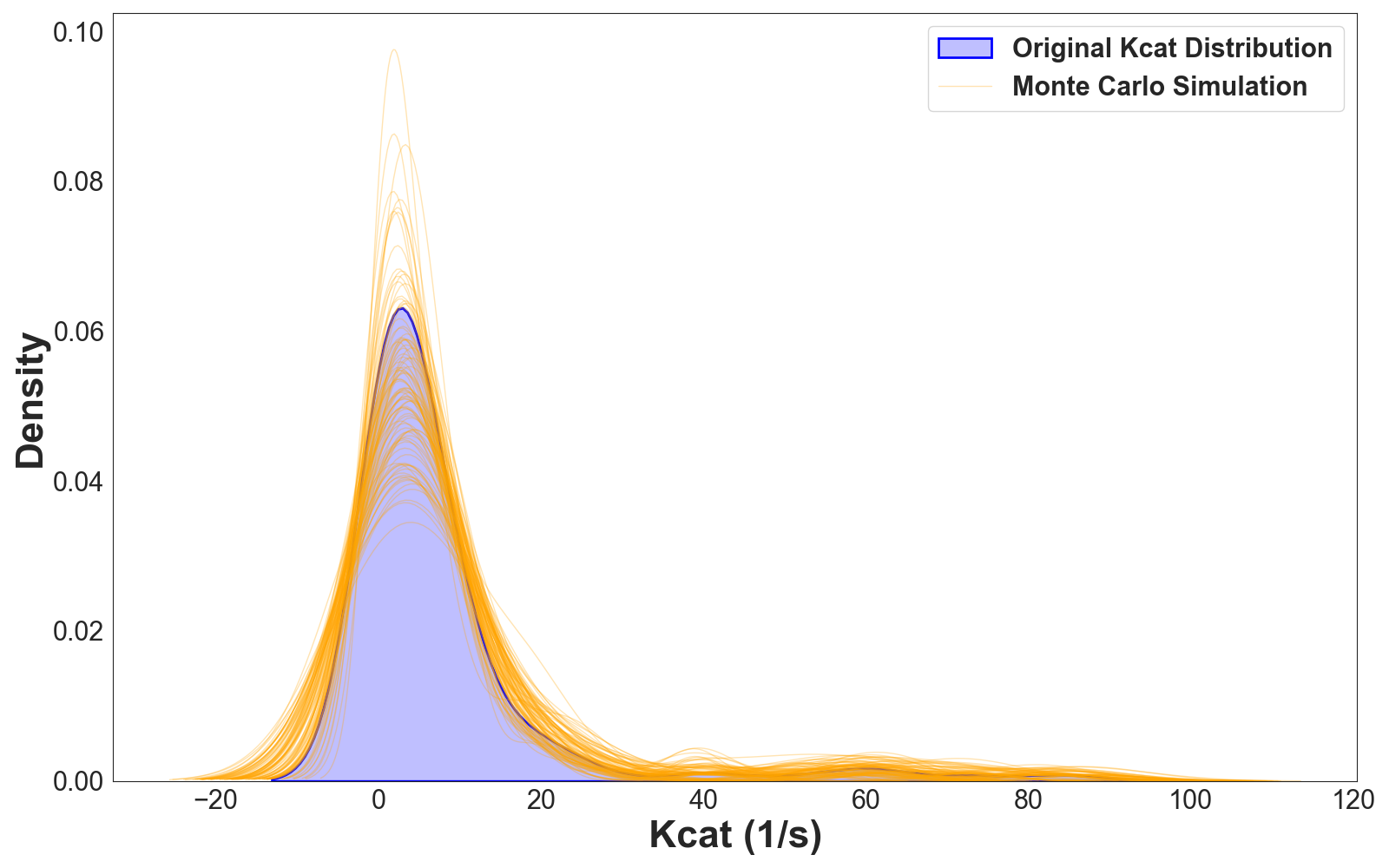


**Fig S1**. Monte Carlo simulations of missing $K_{cat}$ values for 80 reactions in *T. pallidum*. The orange lines represent the density distributions of $K_{cat}$values across 100 Monte Carlo simulations. The original $K_{cat}$ distribution, predicted using DLKcat (2), is shown as the blue-shaded area.


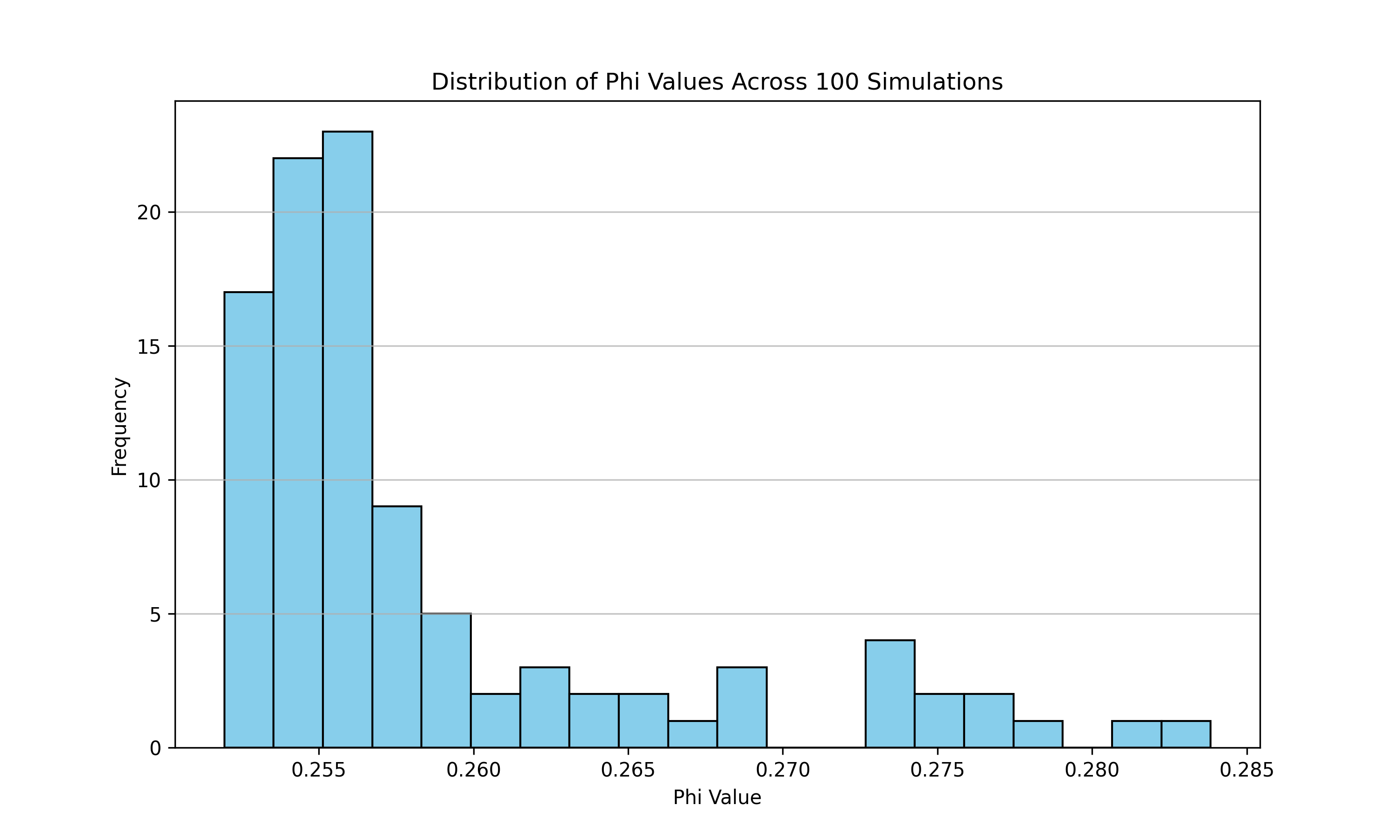


**Fig S2**. Histogram of $\varphi_{MC}$ values from 100 ensemble models. The distribution illustrates the minimum cellular protein content ($\varphi_{MC}$) across simulations, with the lowest value selected to represent *T. pallidum*’s optimized resource allocation.

**References**

1. Han S, Kim D, Kim Y, Yoon SH. 2024. Genome-scale metabolic network model and phenome of solvent-tolerant Pseudomonas putida S12. BMC Genomics 25.

2. Li F, Yuan L, Lu H, Li G, Chen Y, Engqvist MKM, Kerkhoven EJ, Nielsen J. 2022. Deep learning-based kcat prediction enables improved enzyme-constrained model reconstruction. Nat Catal 5:662–672.
